# FAPI-04 PET/CT Using [^18^F]AlF Labeling Strategy: Automatic Synthesis, Quality Control, and *In Vivo* Assessment in Patient

**DOI:** 10.3389/fonc.2021.649148

**Published:** 2021-03-19

**Authors:** Xiao Jiang, Xiaoxiong Wang, Taipeng Shen, Yutang Yao, Meihua Chen, Zeng Li, Xiuli Li, Jiaqi Shen, Ying Kou, Shirong Chen, Xing Zhou, Zhifu Luo, Zhuzhong Cheng

**Affiliations:** ^1^ Radiation Oncology Key Laboratory of Sichuan Province, Sichuan Cancer Hospital, Chengdu, China; ^2^ Institute of Isotope, China Institute of Atomic Energy, Beijing, China

**Keywords:** fibroblast activation protein inhibitor (FAPI)-PET, fibroblast activation protein inhibitor, automatic synthesis, PET molecular imaging, cancer associated fibroblasts, invasive ductal carcinoma

## Abstract

^68^Ga labeled FAPI is the current standard for FAPI-PET, but its batch activity is limited. [^18^F]AlF-NOTA-FAPI-04 is a promising alternative combining the advantages of a chelator-based radiolabeling method with the unique properties of fluorine-18. The objective of this study was to develop a quick automatic method for synthesis of [^18^F]AlF-NOTA-FAPI-04 using a AllinOne synthesis system, and perform PET imaging with [^18^F]AlF-NOTA-FAPI-04 on patients. [^18^F]AlF-NOTA-FAPI-04 was produced, and its quality control was conducted by HPLC equipped with a radioactive detector. [^18^F]AlF-NOTA-FAPI-04 PET/CT imaging was performed in normal BALB/c mice (n = 3) and 4T1 breast cancer models (n = 3) to determine its biodistribution. Then [^18^F]AlF-NOTA-FAPI-04 and ^18^F-fluorodeoxyglucose (FDG) PET/CT imaging were performed in an invasive ductal carcinoma patient (female, 54 years old). The synthesis time of [^18^F]AlF-NOTA-FAPI-04 was about 25 min, and the radiochemical yield was 26.4 ± 1.5% (attenuation correction, n = 10). The radiochemical purity was above 99.0% and was above 98.0% after 6 h. The product was colorless transparent solution with pH value of 7.0–7.5, and the specific activity was 49.41 ± 3.19 GBq/μmol. PET/CT imaging in mice showed that physiological uptake of [^18^F]AlF-NOTA-FAPI-04 was mainly in the biliary system and bladder, and [^18^F]AlF-NOTA-FAPI-04 highly concentrated in tumor xenografts. PET/CT imaging in the patient showed that [^18^F]AlF-NOTA-FAPI-04 obtained high tumor background ratio (TBR) value of 8.44 in segment V and VI, while TBR value was 2.55 by ^18^F-FDG. [^18^F]AlF-NOTA-FAPI-04 could be synthesized with high radiochemical yield and batch production by AllinOne module and show excellent diagnosis performance in cancer patients.

## Introduction

Fibroblast activation protein (FAP) is a widely distributed antigen in epithelial tumor cells, and FAP is closely related to the formation and maintenance of epithelial cell interstitial state, multidrug resistance and immunosuppression (1–3). In normal tissues, FAP is found only in healing wounds, embryonic tissues, and physiological reconstruction organs (4, 5). But FAP was expressed in 90% epithelial tumor tissues and 50–95% tumor stroma (6). Therefore, FAP is a broad-spectrum tumor target with strong specificity and good stability. The tumor treatment strategies using FAP as target, such as fibroblast activation protein inhibitor (FAPI) (7), fibroblast activation protein-activated prodrug (8), CAR-T cell therapy with FAP as the target (9, 10), FAP vaccine (11), have broadened the possibility of cancer treatments, but yet these treatment strategies have not been converted to clinical applications.

As Haberkorn reported, ^68^Ga labeled FAPI shows promising diagnosis efficiency in 30 different types of cancer (12–14). Preliminary results show that FAP targeted positron emission molecular imaging probes accumulate in sarcoma, esophageal cancer, breast cancer, cholangiocarcinoma, and lung cancer with a standardized uptake value (SUV) over 12. In hepatocellular carcinoma, colorectal cancer, head and neck cancer, ovarian cancer, pancreatic cancer, and prostate cancer these probes have a SUV range of 6–12, while in pheochromocytoma, renal cell carcinoma, differentiated thyroid carcinoma, adenoid cystic carcinoma and gastric cancer exhibit maximum SUV of less than 6. Besides, FAP targeted positron emission molecular imaging probes have several advantages comparing with ^18^F-FDG such as unrelated to blood glucose, broader cancer spectrum, higher tumor to background ratio (higher than 3–6) and specificity. Molecular imaging probes targeting FAP are becoming a promising broad cancer spectrum PET tracer.

Currently reported research on molecular imaging probes targeting FAP are using a ^68^Ga labeling strategy (15, 16). However, applications of ^68^Ga labeling FAPI is limited due to relatively short half-life (67.7 min) of ^68^Ga compared with ^18^F, high cost, and low production batch yield of the ^68^Ge/^68^Ga generator. The most widely used PET radionuclide is ^18^F. ^18^F shows several physico-chemical advantages over ^68^Ga especially after McBride developed [^18^F]AlF labeling strategy, which combines the advantages of chelator-based labeling method with unique properties of ^18^F (17–19). Pauwels indicated [^18^F]AlF-NOTA-Octreotide represents a clinical alternative for gallium-68 labeled somatostatin analog PET (*eg.* [^68^Ga]Ga-DOTA-TATE) (20). These promising results make [^18^F]AlF-NOTA-FAPI-04 an ideal alternative candidate for [^68^Ga]Ga-NOTA-FAPI-04.

The aim of this work is to develop a routine clinical production on Trasis AllinOne automated radiosynthesizer platform to produce [^18^F]AlF-NOTA-FAPI-04 with a high radiochemical yield and high specific activity. The validation of the analytical procedures and quality control of [^18^F]AlF-NOTA-FAPI-04 are described. [^18^F]AlF-NOTA-FAPI-04 PET/CT was studied in normal BALB/c mice and 4T1 breast cancer models compared with pathology results of tumor tissues. Furthermore, we perform [^18^F]AlF-NOTA-FAPI-04 PET/CT scan in an invasive ductal carcinoma patient.

## Materials and Methods

### General

Reagents and solvents were purchased from Sigma (Sigma-Aldrich, MO, USA) without further purification if not mentioned. The precursor, NOTA-FAPI-04, was obtained from Paite (Paite Biotech, Beijing, China). Isoflurane was obtained from Shandong Keyuan. Cartridges were purchased from Waters (Waters, MA, USA). Before use, QMA cartridge was flushed with 10 ml of potassium carbonate followed by 40 ml water. Millex-GS syringe filter units were purchased from Millipore (Millipore, MA, USA).

Animal experiments were conducted in compliance with the guidelines for the care and use of research animals established by Sichuan Cancer Hospital Ethic Committee (Permit Number: SCCHEC-04-2020-001). 4-wk-old female BALB/c mice were obtained from Beijing Huabukang.

### Auto Radiosynthesis

**Figure 1:**
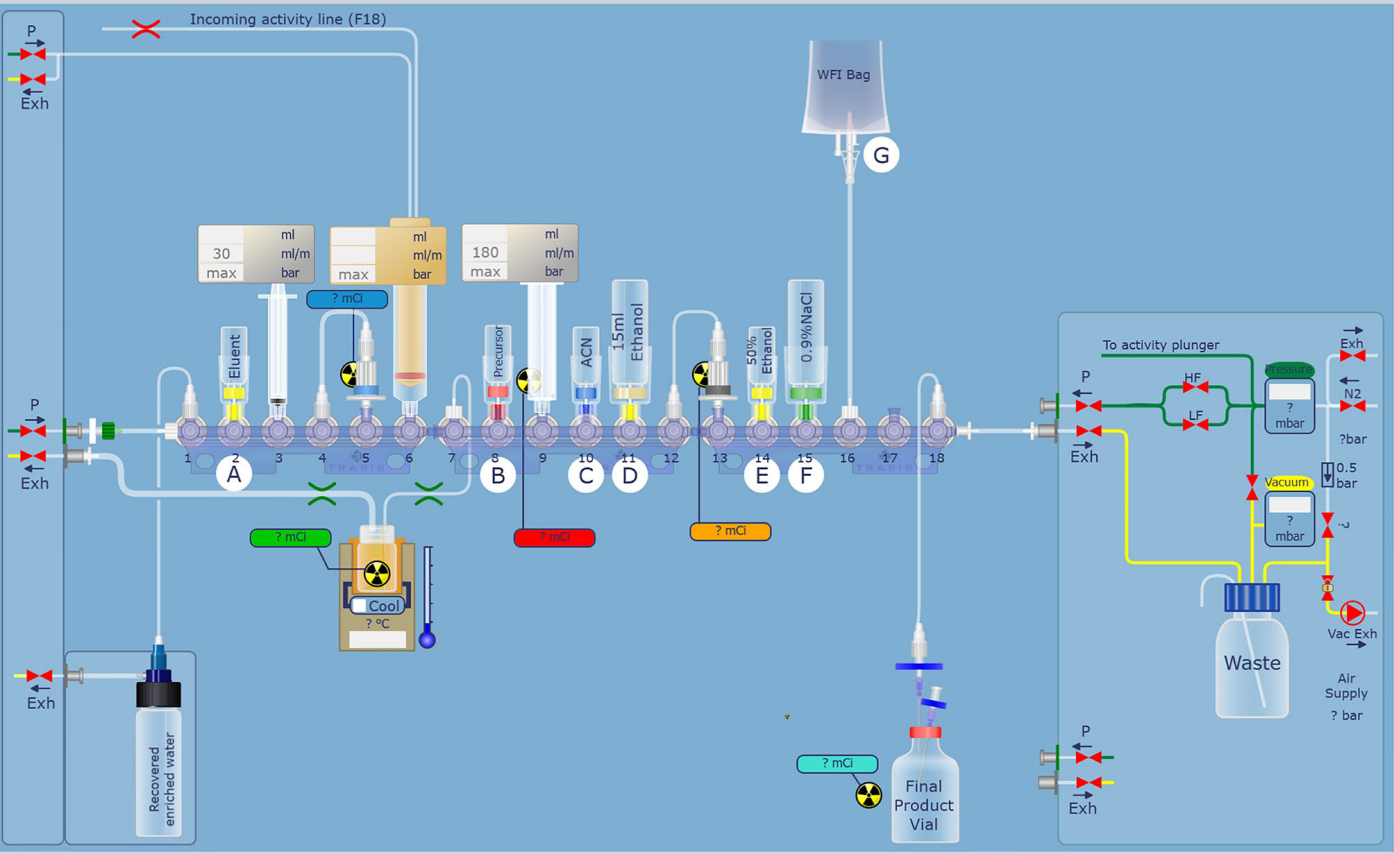
Modified hardware and reagent kits for [^18^F]AlF-NOTA-FAPI-04 based on AllinOne synthesis system.

For automatic synthesis of [^18^F]AlF-NOTA-FAPI-04, AllinOne module (Trasis, Ans, Belgium) was used and modified (**Figure 1**). Synthesis software was programmed in Trasis Suite Software Version 2.30 (Trasis, Ans, Belgium). [^18^F]fluoride was produced on site using Sumitomo HM-10 cyclotron system (Sumitomo Heavy Industries, Tokyo, Japan) by irradiation of [^18^O]H_2_O with 10-MeV protons.

**Figure 2:**
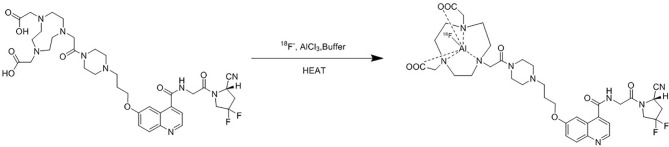
Synthesis of [^18^F]AlF-NOTA-FAPI-04.

After the irradiation, [^18^F]fluoride was transferred to the collection tube and then passed through a Sep-Pak light QMA cartridge. The trapped [^18^F]fluoride was eluted with 1.0 ml saline and then 2.5 ml acetonitrile was added. The mixture was dried up under vacuum condition. 0.15 mg NOTA-FAPI-04 in 2.0 ml anhydrous acetonitrile, 4.0 ml acetate buffer (pH = 4), and 10 μl 1 mg/ml aluminum chloride solution were added to the dried reactor and the mixture was heated at 130°C for 8 min (**Figure 2**). After cooling to room temperature, the reaction mixture was diluted to 8 ml with acetonitrile and then transferred to a pre-conditioned HLB cartridge. 10 ml 5% ethanol solution was passed through the HLB cartridge containing crude product to remove the impurities. The purified product was collected with 0.5 ml anhydrous ethanol, and then passed through a sterile 0.22 μm filter with 10 ml saline containing 0.1 ml 100 mg/L ascorbic acid solution.

### Quality Control

Analytical HPLC (Shimadzu LC-15, Suzhou, China) analysis was used for the quality control of the final product [^18^F]AlF-NOTA-FAPI-04, equipped with a UV/Vis detector preset to 220 nm, an analytical C18 column (Shimadzu WondaSil, 4.6 × 250 mm, 5 μm) and a radioactive detector (Eckert & Ziegler, GA, USA). The column flow rate was 1 ml/min and was kept at approximately room temperature. The samples were eluted with mixtures of mobile phase A: acetonitrile with 0.1% (*v:v*) trifluoroacetic acid (TFA) and water with 0.1% (*v:v*) TFA as mobile phase B. The elution gradient was: 0–30 min: from 8% A to 35% A. The identity of [^18^F]AlF-NOTA-FAPI-04 is confirmed using the validated radioHPLC method by determining the relative retention of the principal peak in the radiochromatogram relative to the NOTA-FAPI-04 peak obtained with the reference solution using the UV/VIS detector.

pH values of the final product were measured with a pH strip. The final product was tested for microbiological contamination and bacterial endotoxins in the department of clinical laboratory of Sichuan Cancer Hospital. [^18^F]AlF-NOTA-FAPI-04 was stored in room temperature and final formula for 6 h, and then radiochemical purity of [^18^F]AlF-NOTA-FAPI-04 was tested using HPLC. The radionuclide purity was determined by gamma spectrometry (Elisya-Raytest, Straubenhardt, Germany).

37 MBq [^18^F]AlF-NOTA-FAPI-04 was added into 10 ml centrifuge tube containing 3 ml n-octanol and 3ml phosphate buffer solution (pH = 7.4). After sealing, the tube was fully whirled for 5 min and then centrifuged at 2,000 revolutions per min for 5 min. 0.5 ml solution of both organic phase and water phase was added into clean tube to measure the radioactivity counts in gamma counter (Wizard^2^ 2470, Perkin-Elmer, MA, USA), respectively. Octanol water partition constant was calculated by dividing radioactivity counts of organic phase by radioactivity counts of water phase.

### Animal Studies

4T1 breast cancer cells was obtained from National Collection of Authenticated Cell Cultures (NCACC), and maintained in RPMI1640 medium (Cellgro, Herndon, VA, USA) supplemented with 10% fetal bovine serum and a mixture of 1% penicilium/streptomycin (Cellgro, Herndon, VA, USA). All experiments with cell lines were conducted within 6 months after obtaining from NCACC. Cell line authentication was done in Sichuan Cancer Hospital. We used short tandem repeat (STR) profiling for characterization and authentication of cell lines. Cells were grown in humidified incubator at 37°C with 5% carbon dioxide atmosphere. Exponentially growing cells were harvested with 0.25% (w/v) trypsin-0.53 mM EDTA solution, and suspended in phosphate buffer saline.

4-wk-old female BALB/c mice were implanted subcutaneously with 1.5 × 10^10^/L 4T1 breast cancer cells in the left hind limb and were used when the tumors reached approximately 0.7 cm in diameter. Normal female BALB/c mice (n = 3) and 4T1 breast cancer rodent models (n = 3) were injected with 7.4 MBq [^18^F]AlF-NOTA-FAPI-04 *via* tail vein under anesthesia (2.5% isoflurane in oxygen at 1.5 L/min flow rate) and kept under anesthesia during the protocols. PET/CT imaging was performed 1 h after radiopharmaceutical administration on a Biograph mCT scanner (Siemens, Erlangen, Germany). Following non-contrast-enhanced low-dose CT (50 keV, 10 mAs; reconstructed to a slice thickness of 1 mm), PET was acquired in 3-D mode (matrix 200 × 200) for 8 min. Attenuation correction was performed using the non-enhanced low-dose CT data. Reconstruction was performed with an ordered subset expectation maximization (OSEM) algorithm (three iterations, 21 subsets).

Mice were euthanized 24 h after tracer injection. Tumors were removed and dissected for paraffin sections, which were examined by hematoxylin and eosin (H&E) stained histopathology and FAP immunohistochemical (IHC) staining. For IHC analysis, 4 μm thick sections were deparaffinized with xylene, and washed with ethanol. Sections were cooled for 20 min then incubated 10 min with 3% H_2_O_2_ to quench endogenous peroxidase activity. Blocking was performed using serum-free protein block, Dakocytomation (Carpenteria, CA, USA), for 30 min. The sections were pretreated with an EDTA buffer saline solution, microwaved for 20 min, and then incubated with an anti-FAP antibody (LS-A8023; polyclonal, 1:100 dilution, LifeSpan BioSciences, WA, USA) for 1 h at room temperature. The diaminobenzidine complex was used as a chromogen.

### Patient and PET/CT Procedure

A 54-year-old woman had right breast modified radical mastectomy for invasive ductal carcinoma (IDC) 4 years ago. Liver metastases were found by CT scan 4 weeks ago. ^18^F-FDG and [^18^F]AlF-NOTA-FAPI-04 PET/CT were performed to evaluate the potential lesions in other locations. [^18^F]AlF-NOTA-FAPI-04 PET/CT imaging was approved by the Sichuan Cancer Hospital Ethic Committee (Permit Number: SCCHEC-04-2020-001), and informed consent was obtained from the patient before PET/CT scan.

296 MBq (8 mCi) [^18^F]AlF-NOTA-FAPI-04 was infused intravenously over 2 min. The whole-body PET/CT imaging (2 min per table position) was performed 1 h after administration on the same Biograph mCT scanner. Following non-contrast-enhanced low-dose CT (140 keV, 42 mAs; reconstructed to a slice thickness of 8 mm), PET was acquired in 3-D mode (matrix 200 × 200) for 14 min. Attenuation correction was performed using the non-enhanced low-dose CT data. Reconstruction was performed with an ordered subset expectation maximization (OSEM) algorithm (three iterations, 21subsets). ^18^F-FDG PET/CT procedure follows the routine clinical pathway.

## Results

### Radiopharmaceutical

The procedures for synthesis of [^18^F]AlF-NOTA-FAPI-04 were performed and validated by ten validation runs using protocols mentioned above. Ten batches of [^18^F]AlF-NOTA-FAPI-04 were successfully synthesized with a final radioactivity of 9.095 ± 0.587 GBq at end of synthesis with a decay-corrected radiochemical yield of 26.4 ± 1.5%. Total synthesis time was about 25 min, starting from [^18^F]F^-^ transfer to the QMA cartridge, to obtain the purified product [^18^F]AlF-NOTA-FAPI-04.

### Quality Control

**Figure 3:**
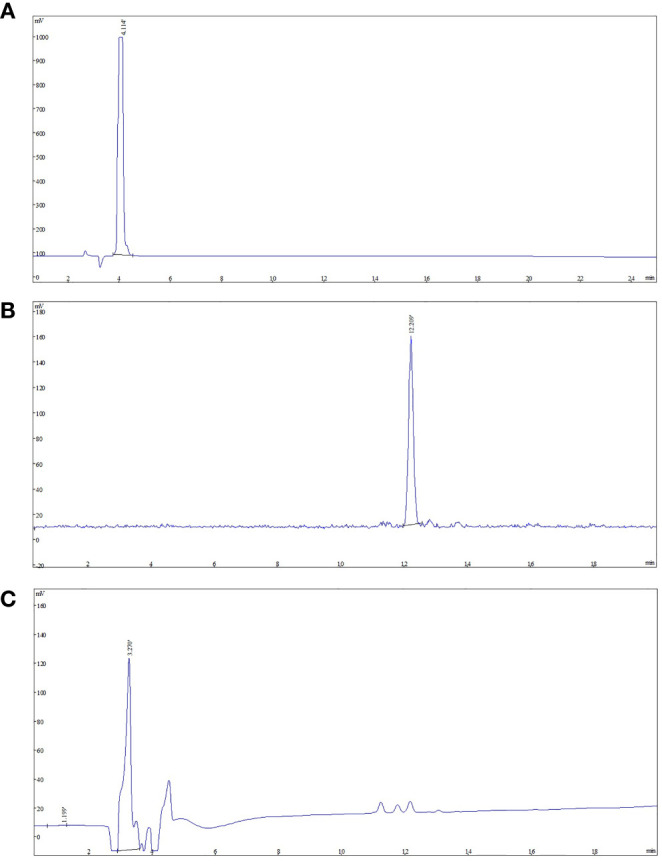
The analytic HPLC chromatogram of purified [^18^F]AlF-NOTA-FAPI-04, UV absorbance at 220 nm. The UV absorbance and radioactive signals were both transformed into electrical signals. **(A)** UV absorbance of NOTA-FAPI-04 precursor. **(B)** The chromatogram of [^18^F]AlF-NOTA-FAPI-04 measured by radioactive detector. **(C)** The chromatogram of [^18^F]AlF-NOTA-FAPI-04 measured by UV detector.

The final product was confirmed by comparing product with NOTA-FAPI-04. The analytical HPLC chromatograms (**Figure 3**) showed that the retention time of NOTA-FAPI-04 was about 4 min in UV/Vis chromatogram. The retention time of [^18^F]AlF-NOTA-FAPI-04 was about 12 min in radiochromatogram meanwhile retention time of the residual unreacted NOTA-FAPI-04 was 4 min in UV/Vis chromatogram. Only one radioactive peak was detected, which suggested the radiochemical purity of product was almost 100% (**Figure 3**). The radionuclide identity (Fluorine-18) was confirmed using the approximate half-life determination test.

The apparent molar activity, based on the amount of [^18^F]AlF-NOTA-FAPI-04, NOTA-FAPI-04 and metal complexes of NOTA-FAPI-04, was found to be 49.41 ± 3.19 GBq/μmol. The pH of the final product was found to be consistently seven. All three batches were found to be sterile and were complying with Ch. P. requirements for bacterial endotoxins.

Octanol water partition constant (*P*) of [^18^F]AlF-NOTA-FAPI-04 was about 5,830 (log*P_O/W_* = 3.766) indicating [^18^F]AlF-NOTA-FAPI-04 displays high lipophilicity. The stability of [^18^F]AlF-NOTA-FAPI-04 in the formulation solution was determined by Analytical HPLC. The results showed the long-term (8 h after synthesis) radiochemical purity of [^18^F]AlF-NOTA-FAPI-04 in final injection formula at room temperature did not show any decomposition. There was no increase of chemical or radiochemical impurities.

### Animal Studies

**Figure 4:**
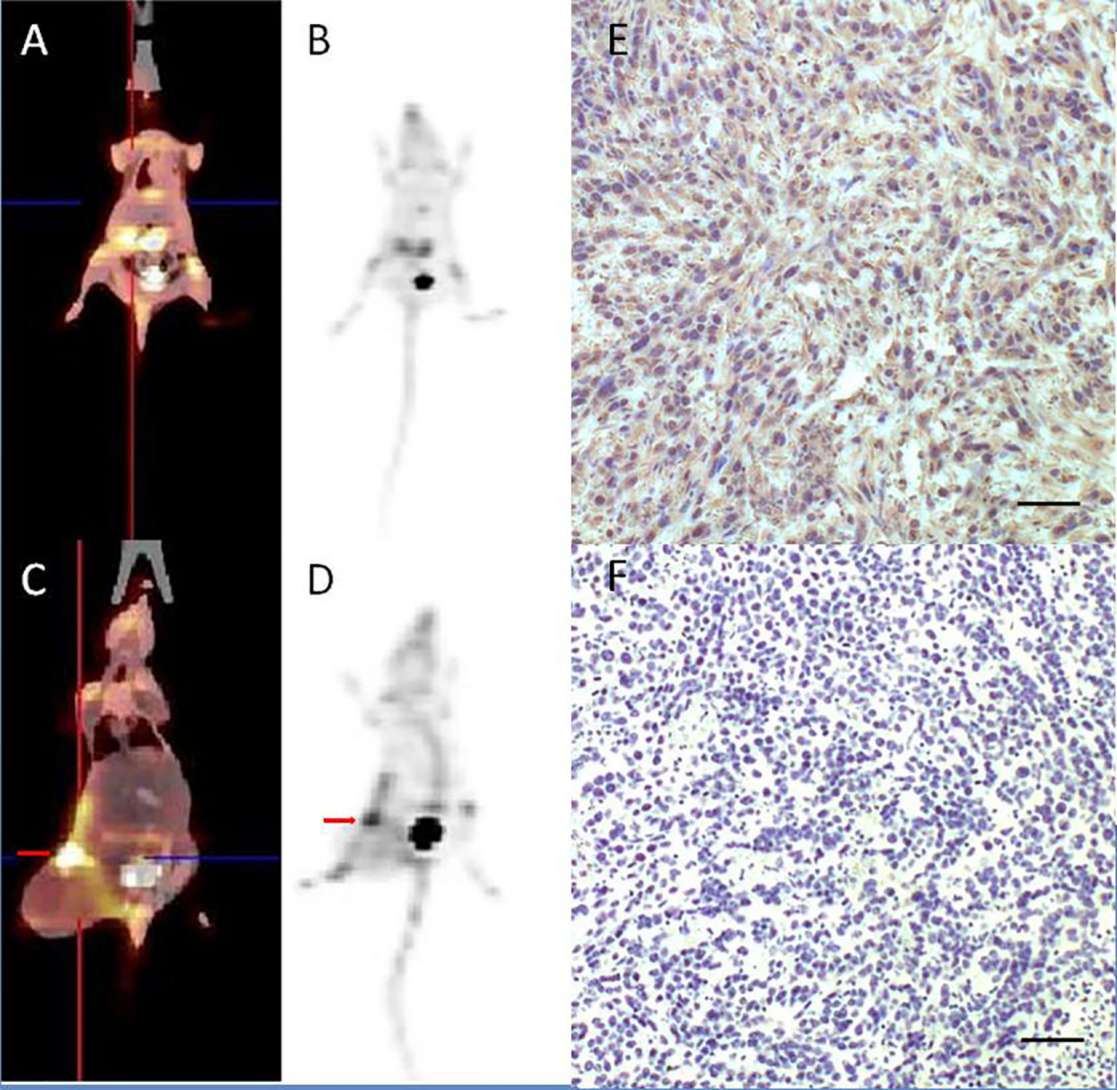
[^18^F]AlF-NOTA-FAPI-04 PET/CT imaging of normal BALB/c mice **(A, B)** and 4T1 breast cancer models (**C, D**, red arrow indicates tumor). FAP IHC staining of 4T1 tumor tissue **(E)** showed 4T1 tumor tissue with high FAP expressing and control group without anti-FAP antibody **(F)** showed there is no false positive in FAP IHC staining results. Scale bar, 100 μm.

The uptake of [^18^F]AlF-NOTA-FAPI-04 as determined by PET/CT imaging and FAP IHC staining is summarized in **Figure 4**. For normal BALB/c mice, high uptake of [^18^F]AlF-NOTA-FAPI-04 was seen in biliary system and bladder. Tracer concentration in blood and other organs was generally low (SUV <0.6) at 1 h post-injection, and only limited defluorination was observed (bone SUV_max_ 0.5 at 1 h post-injection). For 4T1 breast cancer models, 4T1 tumor tissue showed FAP expressing and high uptake was seen in FAP-expressing 4T1 tumor and bladder (4T1 tumor SUV_max_ 5.7).

### PET/CT Imaging

**Figure 5:**
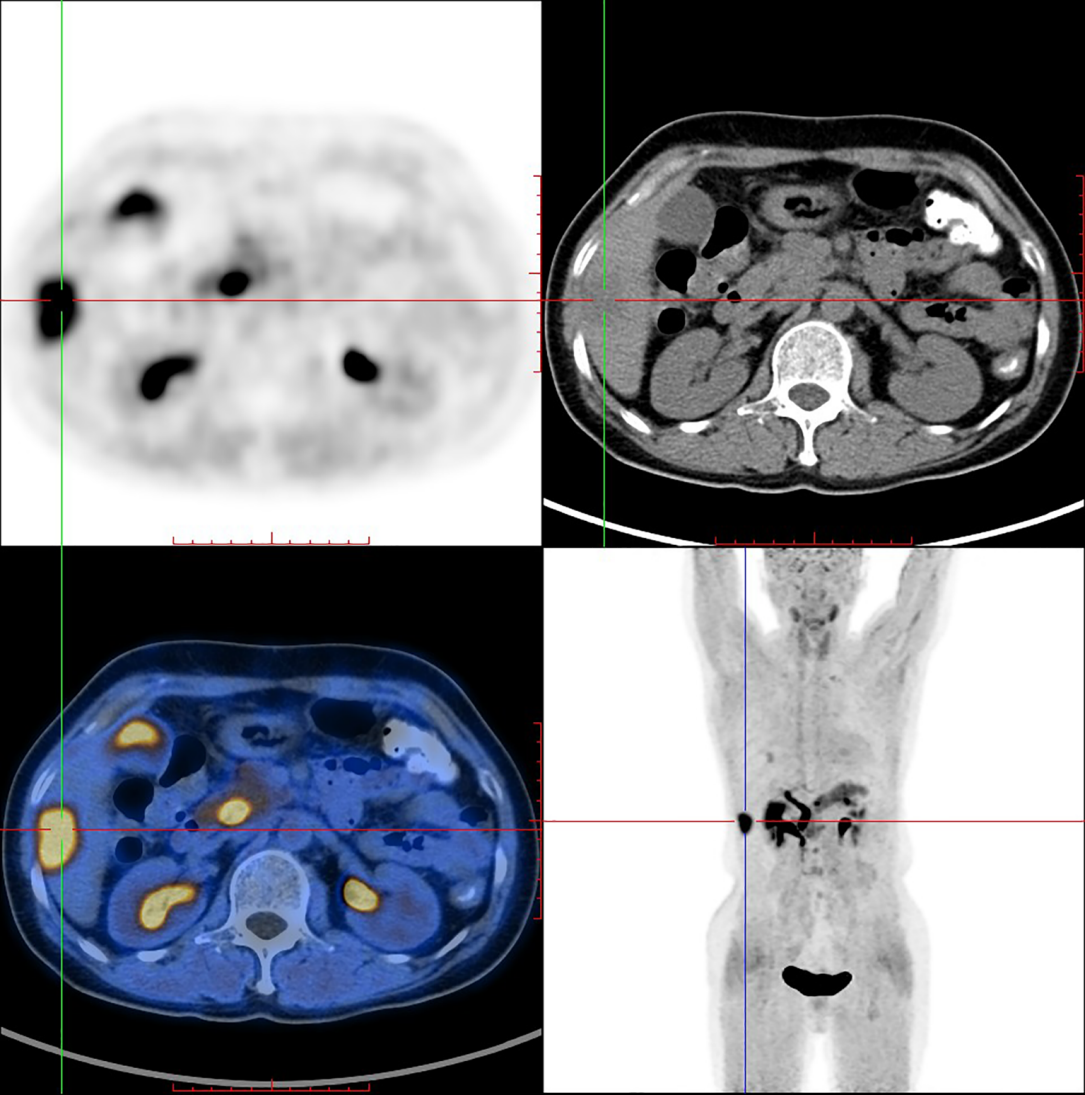
[^18^F]AlF-NOTA-FAPI-04 PET/CT imaging of a female IDC patient (54 y).

PET/CT imaging of [^18^F]AlF-NOTA-FAPI-04 in a female IDC patient was shown in **Figure 5**. Physiological uptake of tracer was seen in bilateral submandibular glands, thyroid, biliary system, pancreas and bladder, and [^18^F]AlF-NOTA-FAPI-04 highly concentrated in liver segments V and VI (SUV _max_ = 10.8) with high tumor background ratio (TBR) value of 8.44.

**Figure 6:**
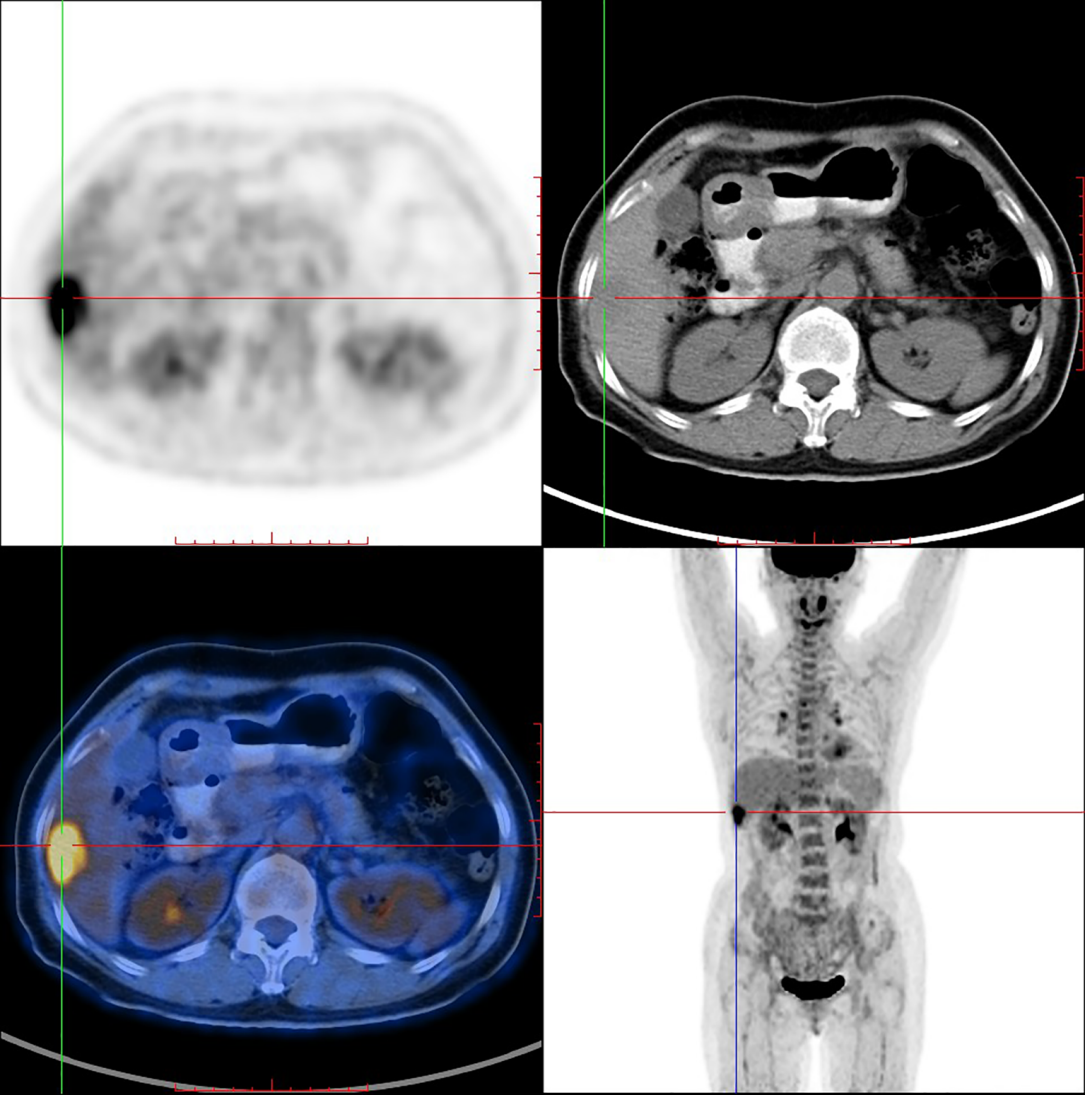
^18^F-FDG PET/CT imaging of the same female IDC patient (54 y).

PET/CT imaging of [^18^F]F-FDG in the same female IDC patient was shown in **Figure 6**. PET/CT imaging showed that [^18^F]F-FDG highly concentrated in the same liver segments V and VI (SUV _max_ = 6.6) with high TBR value of 2.55.

## Discussion

FAP is a promising target for cancer molecular targeting therapy which was widely distributed in the tumor stroma. Quinolines based FAPIs could bind and internalize with FAP catalytic domain (21). Haberkorn et al. reported ^68^Ga labeled FAPI showed promising diagnosis efficiency in 30 different types of cancer with better TBR comparing with ^18^F-FDG (12–14). However, applications of ^68^Ga labeling FAPI are limited due to relatively short half-life (67.7 min) of ^68^Ga compared with ^18^F, high cost and low production of ^68^Ga which only provide for 2–3 patients in one batch. Among all FAPI tracers, ^68^Ga-FAPI-04 was the most studied and reported PET molecular imaging probe. It’s convenient to make comparisons with other studies if we chose FAPI-04 to label. Inspired by [^18^F]AlF labeling strategy and the fact that [^18^F]AlF-NOTA-Octreotide emerged as a clinical alternative for gallium-68 labeled somatostatin analog PET, we aimed to develop a method for the production of [^18^F]AlF-NOTA-FAPI-04.

We successfully described an automated synthesis method of [^18^F]AlF-NOTA-FAPI-04 using AllinOne module with high batch radioactivity (9.095 ± 0.587 GBq) and radiochemical yield (26.4 ± 1.5%), allowing more injections in one production comparing with ^68^Ga labeled FAPI probes. Routine clinical automated production allows for higher batch radioactivity, higher radiochemical yield, higher reliability and less subjective factors. Chelator based radiolabeling methods required metal-free conditions to get high efficiency especially for [^18^F]AlF labeling strategy. Several measures were implemented to avoid interference of metal ions. We used high purity reagents for radiolabeling and plastic tools instead of metal tools. Cartridge purification is used in our production instead of HPLC for it is more simple and reliable in automatic synthesis. Critical parameters for efficient [^18^F]AlF-labeling are the ^18+19^F^−^-to-Al^3+^ ratio and the chelator-to-Al^3+^ ratio in the labeling reaction mixture (22). We opted to use 0.15 mg NOTA-FAPI-04 precursor and 0.01 mg AlCl_3_ for radiolabeling. pH value is another critical parameter for efficient [^18^F]AlF-labeling. We chose 4.0 ml acetate buffer to get the optimal pH (pH = 4) for [^18^F]AlF radiolabeling of [^18^F]AlF-NOTA-FAPI-04. Ascorbic acid was added to prevent radiolysis to get a high radiochemical yield, and it can be only added at the last step as it could cause a drop in pH of the reaction mixture.

The retention time of [^18^F]AlF-NOTA-FAPI-04 was about 12 min in radiochromatogram, meanwhile retention time of the residual unreacted NOTA-FAPI-04 was 4 min in UV/Vis chromatogram. Only one radioactive peak was detected, which suggested the radiochemical purity of product was almost 100%. It is reported the formation of two stereoisomers of [^18^F]AlF-NOTA-Octreotide was observed (23) and the isomerization of macrocyclic bifunctional chelator metal complexes was described before (24–26). This isomerization did not happen in synthesis of [^18^F]AlF-NOTA-FAPI-04.

Biodistribution studies in FAP positive tumor mice showed high specific uptake of [^18^F]AlF-NOTA-FAPI-04 in the tumor and FAP expressing tissues with little or no *in vivo* defluorination. Uptake of background tissue and bone was low, resulting in high contrast images and indicating limited defluorination. These preclinical results supported translation to clinical evaluation.

Recently, Giesel et al. published the results of the first human study comparing [^18^F]AlF-FAPI-74 with ^68^Ga-FAPI-74 for the detection of lung cancer (27, 28). High tumor uptake and contrast were reported. In this study, optimal TBR with limited noise images were obtained 1 h after injection in an IDC patient. The time-dependent biodistribution of [^18^F]AlF-NOTA-FAPI-04 in IDC patient was nearly identical to other FAPI based FAP-PET. [^18^F]AlF-NOTA-FAPI-04 PET/CT imaging an IDC patients showed high tumor uptake, high TBR, and no evidence of *in vivo* defluorination compared with ^18^F-FDG. However, prospective clinical study that compares [^18^F]AlF-NOTA-FAPI-04 directly with [^68^Ga]Ga-NOTA-FAPI-04 in patients is still needed.

It is well expected as FAPI-02/04/46/74 shares a very similar biodistribution and tracer kinetics, therefore radiation burden of [^18^F]AlF-NOTA-FAPI-04 could be similar to other positron emission FAPI tracers. We guess [^18^F]AlF-NOTA-FAPI-04 has a mean normalized effective dose of 1.4–1.7 mSv per 100 MBq as ^18^F-FAPI-74 has a dose of 1.4 mSv per 100 MBq, and ^68^Ga-FAPI-74 has a dose of 1.6 mSv per 100 MBq, which is lower than PET/CT scans with ^18^F-FDG (2.0 mSv per 100 MBq) (27). Radiation dosimetry of [^18^F]AlF-NOTA-FAPI-04 will be investigated in future work.

## Conclusion

^68^Ga labeled FAPI is the current standard for FAPI-PET, but its batch activity is limited. [^18^F]AlF-NOTA-FAPI-04 is a promising alternative combining the advantages of a chelator-based radiolabeling method with the unique properties of fluorine-18. We developed an automatic synthesis that allows quick, high yield, and high batch activity production of [^18^F]AlF-NOTA-FAPI-04. Furthermore, the production process and quality control developed for [^18^F]AlF-NOTA-FAPI-04 are easily implementable in a clinical setting. [^18^F]AlF-NOTA-FAPI-04 showed high *in vivo* stability and favorable pharmacokinetics with high and specific accumulation in both 4T1 tumor bearing rodent models and an IDC patient.

## Data Availability Statement

The raw data supporting the conclusions of this article will be made available by the authors, without undue reservation.

## Ethics Statement

The studies involving human participants were reviewed and approved by the Sichuan Cancer Hospital Ethic Committee. The patients/participants provided their written informed consent to participate in this study. The animal study was reviewed and approved by Sichuan Cancer Hospital Ethic Committee.

## Author Contributions

XJ, XW, TS, YY, MC, ZL, XL, JS, YK, SC, ZFL, and ZC conceived and designed the study and helped to draft the manuscript. XJ, YY, and XZ performed the data collection. XJ and JS performed the statistical analysis. All authors contributed to the article and approved the submitted version.

## Funding

This work has been supported by the Sichuan Science and Technology Project (grant numbers 2019YJ0574 and 2020YFS0421).

## Conflict of Interest

The authors declare that the research was conducted in the absence of any commercial or financial relationships that could be construed as a potential conflict of interest.
